# The ageing neuromuscular system and sarcopenia: a mitochondrial perspective

**DOI:** 10.1113/JP271212

**Published:** 2016-05-07

**Authors:** Karolina A. Rygiel, Martin Picard, Doug M. Turnbull

**Affiliations:** ^1^ Newcastle University Centre for Ageing and VitalityNewcastle upon TyneUK; ^2^ Wellcome Trust Centre for Mitochondrial ResearchNewcastle upon TyneUK; ^3^ Division of Behavioral Medicine, Department of Psychiatry, College of Physicians and Surgeons, Columbia UniversityColumbia University Medical CenterNew YorkNYUSA

## Abstract

Skeletal muscles undergo structural and functional decline with ageing, culminating in sarcopenia. The underlying neuromuscular mechanisms have been the subject of intense investigation, revealing mitochondrial abnormalities as potential culprits within both nerve and muscle cells. Implicated mechanisms involve impaired mitochondrial dynamics, reduced organelle biogenesis and quality control via mitophagy, accumulation of mitochondrial DNA (mtDNA) damage and respiratory chain defect, metabolic disturbance, pro‐apoptotic signalling, and oxidative stress. This article provides an overview of the cellular mechanisms whereby mitochondria may promote maladaptive changes within motor neurons, the neuromuscular junction (NMJ) and muscle fibres. Lifelong physical activity, which promotes mitochondrial health across tissues, is emerging as an effective countermeasure for sarcopenia.

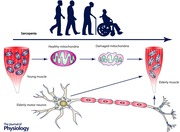

## Introduction

Life expectancy in the modern world is rapidly increasing but the quality of life is often compromised towards the end of the mature years. A determinant of functional capacity and autonomy is the integrity of the neuromuscular system, which wires the brain and skeletal muscles via motor neurons and the neuromuscular junction. With age, different components of this system may fail, leading to loss of muscle mass and function, and decreased ability to remain physically active. This review will focus on structural and functional features of muscle ageing and, more specifically, on the role of mitochondria in relation to various maladaptive mechanisms contributing to age‐related neuronal and muscular decline. We also discuss the role of exercise as currently the most successful intervention against age‐related neuromuscular degeneration.

## Sarcopenia

It has been known for a few decades that skeletal muscle structure and function deteriorate with age. Muscle mass decreases, reflected in both the reduced cross‐sectional area of individual myofibres and of the total number of fibres (Lexell & Taylor, 1991). This is associated with reduced strength and physical performance, culminating in the frailty syndrome and falls that lead, in extreme cases, to immobility and loss of autonomy (Visser & Schaap, 2011; Landi *et al*. 2012). Sarcopenia is defined as a measurable level of muscle wasting based on lean mass, grip strength and gate speed (Cruz‐Jentoft *et al*. 2010). Although sarcopenia eventually affects everyone, the time point at which it begins shows substantial inter‐individual variability – some experience only modest changes in old age, while others become severely disabled as early as in their seventh decade of life.

The causes of sarcopenia are still debated but it has become apparent that involvement of both the muscle itself and the innervating nerve play a role. Voluntary movement is a highly specialized and orchestrated function of the body that requires efficient communication between the nervous and muscular systems. A decision to move triggers excitation of the upper motor neurons residing in the motor cortex of the brain. These cells transmit an action potential to the lower motor neurons within the posterior area of the spinal cord. The electrical impulse then spreads from the cell body of a motor neuron through its axon to the neuromuscular junction (NMJ), a specialized synapse between the neuron and the muscle. There, the incoming neuronal action potential is transmitted by the neurotransmitter acetylcholine to the sarcolemma where it triggers depolarization of the myofibre and initiates muscle contraction.

Because the maintenance of muscle mass requires normal innervation and regular activation, malfunction of any of these elements can lead to the muscle deterioration. The exact causes underlying the age‐related changes in the neuromuscular system are still unknown but there is evidence that mitochondria, a critical cellular organelle involved in energy production and cellular signalling, may be either a primary trigger or at least an important player in this process. Here we will discuss known mitochondrial changes and their potential role in the development of sarcopenic phenotype.

## Mitochondrial structure and functions

### Mitochondrial DNA and the respiratory chain

Mitochondria contain their own genetic material, the mitochondrial DNA (mtDNA). The mtDNA differs from the nuclear DNA (nDNA) in multiple aspects: it is short (∼16.6 kb) and circular, uniparentally inherited from the mother, contains little (<2%) non‐coding regions, and is unprotected by histones. In addition, multiple mtDNA copies exist within a single mitochondrion, and multiple mitochondria reside in the cytoplasm of each cell, creating the possibility of heteroplasmy where different mtDNAs (e.g. mutant and normal) can coexist in a single cell (Li, Schroder *et al*. 2015). Despite its small size, mtDNA is essential for cellular function. It contains 37 genes encoding two ribosomal RNA (rRNA) and 22 transfer RNA (tRNA), which are essential to the translation of its 13 messenger RNA (mRNA)‐coding genes (Taylor & Turnbull, 2005). The resulting polypeptides combine with a number of nuclear‐encoded subunits to make up the multiprotein complexes I, III, IV and V of the mitochondrial respiratory chain. Only complex II is entirely encoded by the nuclear genome.

Attesting to the significance of mtDNA gene products, maternally inherited mtDNA mutations result in mitochondrial disease that is often lethal or severely debilitating, affecting primarily high energy demanding tissues such as the central nervous system and the muscle (Taylor & Turnbull, 2005). In addition, the mtDNA can accumulate defects (point mutations, deletions, duplications) with ageing, which are proposed to cause of age‐related functional decline systemically (Wallace, 2013). This includes the neuromuscular system, as discussed later in this article.

### Mitochondrial dynamics

Mitochondria are highly dynamic organelles. Not only do they change shape and travel significant distances but they also fuse with or fragment from the neighbouring mitochondria (Westermann, 2010; Archer, 2013). A few proteins are required for mitochondrial dynamics: mitofusin 1 and 2 (Mnf1, Mnf2) and optic atrophy 1 (OPA1) for fusion; and dynamin‐related protein 1 (Drp1), mitochondrial fission factor (Mff) and fission protein 1 (Fis1) for fission (Detmer & Chan, 2007). These processes of fusion and fission allow communication and exchange of matrix content between individual mitochondria, including proteins (Chen *et al*. 2010) and mtDNA (Ono *et al*. 2001).

Normal mitochondrial dynamics is crucial for the health of the mitochondrial population as well as for that of the entire cell and organism (Archer, 2013). Alterations of both fusion and fission processes have pervasive effects on several aspects of mitochondrial function including respiratory capacity, coupling, reactive oxygen species production and apoptotic sensitivity (Picard *et al*. 2013). Overall, excessively fragmented mitochondria tend to exhibit reduced respiratory chain capacity, increased ROS production, and increased susceptibility to the release of mitochondria‐derived activators of caspases. Notably, inhibition of mitochondrial fusion by the removal of Mfn 1/2 in skeletal muscle promotes the accumulation of mtDNA defects and small muscle size (Chen *et al*. 2010).

### Quality control

One of the most efficient methods of recycling of damaged/dysfunctional mitochondria is a specialized type of autophagy called mitophagy. Mitophagy is a process closely linked to mitochondrial dynamics (Youle & van der Bliek, 2012), by which a fragmented (small) mitochondrion becomes encapsulated with a double membrane (isolation membrane, see Fig. 2) to form an autophagosome delivered to a proximal lysosome. Upon fusion with the lysosome, the autophagosome cargo undergoes hydrolytic lysis (Kim *et al*. 2007). Mitophagy is designed to remove entire organelles whereas selected mitochondrial proteins are degraded via one of three systems: the ubiquitin–proteasome pathway (Hershko & Ciechanover, 1992), Lon protease (Bota & Davies, 2002), and mitochondria‐derived vesicles (MDVs) (McLelland *et al*. 2014). Removal of dysfunctional mitochondria by mitophagy is essential for maintaining muscle mass (Masiero *et al*. 2009). Several overlapping pathways thus exist to ensure the maintenance of a healthy pool of mitochondria in different cell types as alterations of these pathways may adversely affect muscle health in ageing.

## Role of the lower motor neuron and the neuromuscular junction in sarcopenia

### Lower motor neuron

**Figure 1:**
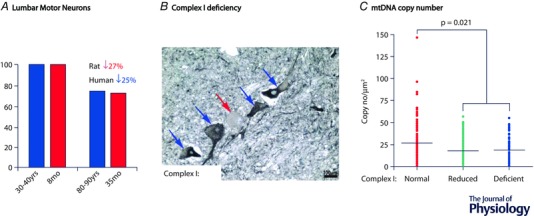
**Lower motor neuron changes with advancing age** *A*, spinal motor neuron population undergoes a similar rate of depletion in ageing in both rats and humans. *B*, some of the remaining motor neurons accumulate somatic mitochondrial damage including severe down‐regulation of mitochondrial respiratory chain complex I, shown here by the absence of labelling for NDUFB8. *C*, it is likely that complex I deficiency is a result of mtDNA depletion in ageing spinal motor neurons.

Neuronal stimulation of the muscle via the NMJ is an essential step for muscle contraction and human movement. Without it the muscle atrophies, losing strength and power (Scelsi *et al*. 1980). It has been reported that the spinal motor neuron population becomes depleted with advancing age (Fig. 1). Stereological analysis of lumbar spinal cords from a rat model of sarcopenia demonstrated 27% reduction in motor neuron pool between young adults and senescent animals (Rowan *et al*. 2012). A similar rate of motor neuron depletion was noted for human post‐mortem samples where 25% loss of these cells occurred between the second and tenth decade of life (Tomlinson & Irving, 1977).

Because muscle fibres cannot remain denervated (Carlson, 2014), the loss of motor neurons promotes lateral sprouting of the neighbouring ones, which reinnervate ‘orphelin’ denervated muscle fibres. This increases the size of the individual motor units, thus elevating the metabolic and biosynthetic burden on a single nerve cell, possibly making it prone to overload‐driven degeneration (Stalberg & Fawcett, 1982; Brown *et al*. 1988). This results in fibre type grouping in the muscle (i.e. the physical clustering of myofibres with the same myosin heavy chain isoform) as multiple muscle fibres are innervated, and fibre‐type dictated by a single motor neuron (Lexell & Downham, 1991; Lexell, 1997).

### Neuromuscular junction

The NMJs have also shown age‐related changes. Data obtained from animal models and humans, and across different muscle groups, are sometimes conflicting. The general consensus, however, is that with ageing the complexity and morphology of the pre‐ and postsynaptic regions become altered, the number of neurotransmitter‐containing synaptic vesicles decreases, and the axonal transport is slower (Jang & Van Remmen, 2011). We now discuss the potential role of mitochondrial defects in NMJ maintenance.

### Mitochondrial deficiency in the ageing neuron

Mitochondria have been implicated in the age‐related alterations of the motor neuron soma as well as the pre‐ and postsynaptic regions of the NMJ. Our recent study in elderly (68–99 years old) post‐mortem spinal cord samples showed a pronounced mitochondrial dysfunction in the lumbar motor neurons (Rygiel *et al*. 2014) (Fig. 1). Around 10% of these cells demonstrated a complete loss of mitochondrial respiratory chain complex I and a further 25% had markedly reduced levels of complex I proteins. Mitochondrial DNA analysis carried out on individual complex I‐deficient motor neurons revealed significantly reduced copy numbers (Fig. 1). Interestingly, the respiratory‐deficient cell bodies were smaller than their unaffected counterparts suggesting mitochondrial deficiency‐driven atrophy (Rygiel *et al*. 2014), not unlike cell atrophy observed in a cytoplasmic hybrid cell of mtDNA heteroplasmy (Picard *et al*. 2014). This acquired mitochondrial dysfunction could originate within the soma of a motor neuron itself, or in the peripheral axonal/dendritic mitochondria, and then be retrograde‐transported back to the soma where it can impact central cellular functions including gene expression.

### Mitochondrial abnormalities in the neuromuscular junction

**Figure 2:**
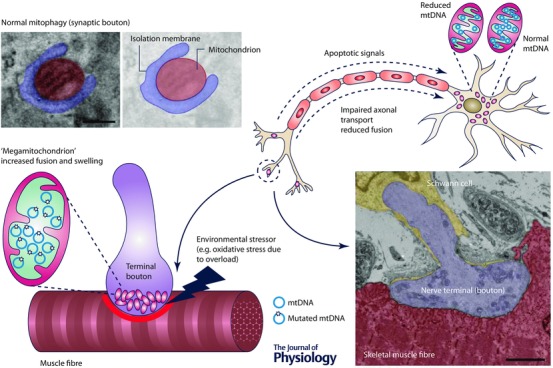
**Proposed mechanism of neuronal mitochondria involvement in sarcopenia** Age‐related increase in oxidative damage or down‐regulation of anti‐oxidant defences leads to mitochondrial damage (swelling, diminished cristae and elevated fusion) in terminal boutons of the neuromuscular junction (NMJ). Reduced fission may prevent mitophagy‐driven clearance of the damaged mitochondria, which accumulate abnormal proteins and mutated mtDNA. These mitochondria become respiratory‐deficient, which leads to inefficient neuromuscular transmission, reduced membrane potential and release of pro**‐**apoptotic factors (e.g. cytochrome *c*). Apoptotic signals are transduced via retrograde transport to the neuronal cell body where they induce apoptosis. Impaired mitochondrial dynamics in the terminal bouton affects axonal transport, and hence distal mitochondria do not mix with their counterparts located in the soma. This potentially results in reduced mtDNA content and down‐regulation of mitochondrial respiratory chain proteins with complex I being affected the earliest. Image at bottom right shows an NMJ from a mouse skeletal muscle (diaphragm) fibre visualized by transmission electron microscopy (TEM). Electron micrograph in the top left corner shows a mitochondrion undergoing mitophagy. Scale bar: 2 μm (NMJ, bottom right); 200 nm (mitophagosome, top left). Images kindly provided by the Basil Petrof Laboratory and the Newcastle University EM Research Services.

Mitochondrial abnormalities within the NMJ may also contribute to impaired signal transduction between the motor neuron and muscle. Indeed, mitochondria exist in presynaptic terminals where they modulate neurotransmitter release (Vos *et al*. 2010). A recent study in aged rats demonstrated substantial changes in the majority of axonal mitochondria residing within the terminal boutons of the tibial nerve (Garcia *et al*. 2013). Electron microscopy analysis revealed unusual features among presynaptic mitochondria: they were swollen, up to 3‐fold larger than their normal counterparts, with ‘hollow’ matrix, virtually devoid of cristae, and with ruptured membranes. A marked proportion of these mitochondria appeared hyperfused, forming gigantic ‘megamitochondria’ with multiple surrounding membrane layers. None of these morphological alterations were observed in the neuronal cell bodies, suggesting specific alterations of NMJ mitochondria (Fig. 2). Indeed, several differences exist between synaptic and non‐synaptic mitochondria, notably in protein composition, which is skewed towards a pro‐fission phenotype (more Drp1, less OPA1 and Mfn1/2) in the mouse brain (Stauch *et al*. 2014). Synaptic mitochondria may also be more prone to mtDNA deletions (Stauch *et al*. 2014). Moreover, calcium‐dense inclusions were reported to be abundant in the aged presynaptic mitochondria, potentially indicating calcium overload (Garcia *et al*. 2013).

A consequence of mitochondrial calcium overload is the release of mitochondria‐derived apoptotic activators such as cytochrome *c* (Brookes *et al*. 2004). Cytochrome *c* was indeed found in the cytosol in the distal portion of motor neurons, suggesting mitochondrial permeability transition and possibly downstream activation of the apoptosome. Caspase 3 was detected in the soma and nuclei of the aged motor neurons as well as the proximal and distal axons, but the activated caspase 3 was clearly associated with vesicles along the microtubule system and colocalized with dynein, implying retrograde transport (Garcia *et al*. 2013) (Fig. 2). The underlying causes of the described mitochondrial pathology and activation of the apoptotic machinery in ageing nerve terminals are unclear but they could be at least partially associated with up‐regulation of the oxidative damage and down‐regulation of the natural cellular anti‐oxidant defences. Studies on superoxide dysmutase 1 (Sod 1) knockout mice revealed a sarcopenic phenotype with the accumulation of abnormal giant mitochondria, higher susceptibility to calcium‐induced mitochondrial permeability transition pore opening and apoptosis, linking oxidative damage with mitochondrial dysfunction and neuromuscular junction denervation (Jang *et al*. 2010).

All of these findings are consistent with the ‘dying back’ phenomenon, where the neuronal damage is inflicted on the axon terminal, causing degeneration of the entire cell. A number of neurodegenerative disorders including motor neuron disease, Alzheimer's disease, Parkinson's disease and glaucoma share this pattern of neuronal degeneration, and therefore it is not surprising that a similar mechanism could be engaged in sarcopenia (Adalbert & Coleman, 2013). Accumulation of abnormal mitochondria primarily in the terminal boutons rather than the neuronal cell bodies can also be explained by the impaired mitochondrial dynamics. A balance between mitochondrial fusion and fission has to be maintained in order to support mitochondrial function and motility. Neurodegenerative diseases such as Charcot–Marie–Tooth 2A (CMT2A) and dominant optic atrophy (DOA) manifest with degeneration of sensory and motor nerves and optic nerve as a direct consequence of mutations in genes encoding mitochondrial fusion proteins Mfn2 and OPA1, respectively (Chan, 2012). It has been proposed that impaired fusion not only leads to reduced function of the fragmented mitochondria but also to accumulation of them in various areas within the neuron resulting in ineffective distribution (Chen & Chan, 2006). Given their potentially skewed protein profile towards fission (Stauch *et al*. 2014), synaptic mitochondria may be particularly vulnerable to factors that impair normal mitochondrial fusion.

It is possible that due to some environmental insult (e.g. oxidative stress) impaired mitochondrial dynamics in the ageing motor neuron leads to dysmorphic and abnormally large mitochondria in terminal NMJ boutons. Because of physical and possibly other constraints, these mitochondria may be unable to move via axonal transport and communicate with other mitochondria, and would escape mitotophagy, failing to be degraded or removed, and thus accumulate protein and mtDNA damage and become dysfunctional. Because of the mitochondria's dynamic role in regulating synaptic function (Sun *et al*. 2013), this would promote dysregulation of neuromuscular transmission, NMJ alteration and ultimately denervation of the associated myofibre (Fig. 2).

## Skeletal muscle

Neuronal dysfunction undoubtedly contributes to age‐related muscle wasting, but the muscle itself also undergoes complex remodelling that exacerbates the sarcopenic phenotype observed in the elderly. The most commonly reported muscle‐specific changes include lower rate of anabolism, reduced regenerative capacity due to the senescence or depletion of the satellite cell pool and higher rate of cell death. The following sections discuss the role of mitochondria in relation to these aspects of age‐related muscle tissue deterioration.

### Skeletal muscle anabolism and catabolism

The major hallmark of the sarcopenic muscle is its reduced size, which can only be explained by a dysregulated protein ‘economy’, namely an imbalance between the rates of protein synthesis (i.e. anabolism) and degradation (i.e. catabolism). These processes are tightly controlled, among other pathways, by insulin signalling via the mammalian target of rapamycin (mTOR) serine/threonine kinase pathway (Bonaldo & Sandri, 2013). Lack of insulin in type I diabetes patients promotes loss of muscle protein content and consequent wasting (Tessari *et al*. 1990). Furthermore, insulin signalling in conjunction with amino acids not only stimulates protein synthesis but also inhibits proteolysis.

As protein synthesis requires energy input, it is not surprising that mTOR also augments mitochondrial function, mitochondrial respiratory chain protein levels and ATP production (Albert & Hall, 2015). Although experimental muscle insulin infusion in both animals and humans resulted in an increased rate of synthesis of mitochondrial proteins including citrate synthase and cytochrome *c* oxidase, it had no effect on structural muscle proteins, including contractile myosin heavy chain, (Boirie *et al*. 2001; Stump *et al*. 2003), indicating that mitochondrial biogenesis and muscle anabolism may be independently regulated.

### Skeletal muscle lipids and metabolism

Insulin resistance is thought to be connected with the intramyocellular lipid accumulation and excess mitochondrial reactive oxygen species in ageing muscle (Anderson *et al*. 2009). Muscle of older people (∼70 year old) has higher lipid content (individual lipid droplets are larger) than muscle of younger people (∼20 year old), and the lipid stores are rarely associated with mitochondria. Physical association of mitochondria with lipid droplets is increased in myofibres after exercise (Tarnopolsky *et al*. 2007), which implies an optimal position for oxidation. Mitochondrial mass is reduced in the old, as a result of fewer individual mitochondria rather than their size, which agrees with reduction in the mitochondrial enzymes (Crane *et al*. 2010). In middle‐aged primates accumulation of larger lipid droplets was also observed together with a shift in fibre type distribution, reduced oxidative phosphorylation capacity and a metabolic shift (increased FAD and NADH levels *in situ*) (Pugh *et al*. 2013). This suggests reduced activity of mitochondrial respiratory chain dehydrogenase enzymes, which may become a limiting factor in aged muscle mitochondria.

The difficulty with interpreting the age‐related lipid accumulation in muscle is that physical inactivity also results in a similar phenomenon. In human diaphragm, compared with normally contracting diaphragm muscle, inactivity during mechanical ventilation exhibited reduced respiratory chain complex IV activity, which was associated with significantly higher intramuscular lipid content manifesting as an increased lipid density as well as droplet volume (Picard *et al*. 2012). Interestingly, lipid accumulation in muscle fibres colocalized with mitochondrial respiratory deficiency measured using histochemical detection of enzymatic activity, indicating a potential causative link between mitochondrial dysfunction and dysregulated lipid metabolism.

### Mitochondrial DNA – genetics and maintenance

Changes in the mitochondrial genome have been implicated in physiological ageing of the majority of organs. In muscle, mtDNA content is inversely related to age (Short *et al*. 2005) and the remaining mtDNA copies acquire rearrangements in an age‐related manner (Meissner *et al*. 2006). Large‐scale mtDNA deletions are most frequently found (Kraytsberg & Khrapko, 2005; Bua *et al*. 2006; Meissner *et al*. 2006), but there have been reports of duplications and triplications detected in muscle from elderly individuals (Tengan & Moraes, 1998).

**Figure 3:**
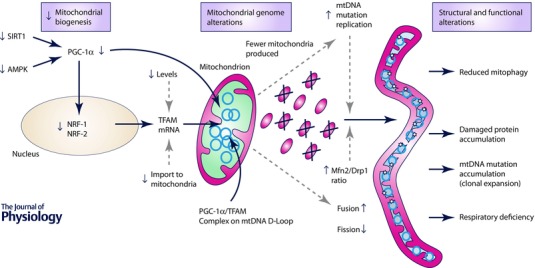
**Proposed mechanism of skeletal muscle mitochondria involvement in sarcopenia** Mitochondrial biogenesis declines with age, dependent and independent of PGC‐1α. Proteins essential for PGC‐1α post‐translational modification, namely SIRT1 and AMPK, are either down‐regulated (SIRT1) or less responsive to activation (AMPK), resulting in depletion of active PGC‐1α pool. Consequently, the downstream nuclear factors NRF‐1 and NRF‐2 are down‐regulated, leading to a reduction in TFAM transcription, and transport of TFAM protein into mitochondria. TFAM and PGC‐1α form a complex, localised to the D‐loop region of mtDNA, which regulates replication and transcription of the mitochondrial genome. Age‐related depletion of both causes reduced nucleic acid and protein turnover, which leads to not only reduced mitochondrial mass but also accumulation of mtDNA mutations and damaged proteins. This induces fusion as a compensatory mechanism to reduce the detrimental consequences of the accumulated defects. Fused mitochondria escape mitophagy and continue accumulating mtDNA (clonal expansion) and protein damage, which ultimately leads to respiratory deficiency.

The origin of mtDNA deletions is still unclear, but it is likely that they are either inherited at low levels from the maternal germline, or acquired somatically early in life. Somatic mutations are formed as a consequence of either replication errors or defective repair of double‐strand breaks (Bua *et al*. 2006; Krishnan *et al*. 2008). At the beginning of this process there are very few mutated molecules present amongst masses of healthy mtDNA counterparts in the cell, but with time, the mutated copies replicate and begin to outnumber normal ones. This process, termed clonal expansion, manifests only in a limited number of single muscle fibres. As a result, the ageing muscle becomes a mosaic of deletion‐loaded and deletion‐free cells (Murphy *et al*. 2012). Interestingly, due to the multiple‐copy nature of mtDNA, the cells are fully respiratory‐functional until they cross a certain threshold of mutated‐to‐healthy molecules. This threshold is tissue and cell type dependent but often only 10% of healthy mtDNA molecules is enough for the cells to maintain normal respiratory functions (Stewart & Chinnery, 2015) (Fig. 3).

Mechanisms behind clonal expansion are still unclear. What is certain is that mitochondrial deletions accumulate in longitudinal segments of individual muscle fibres. Studies on ageing rats have elegantly demonstrated that there is a functional relationship between mitochondrial dysfunction and muscle phenotype. Muscle fibre segments of complete respiratory deficiency due to a high mutation load showed atrophy, splitting and, in some cases, rupturing (Bua *et al*. 2002). However, this phenomenon is relatively rare in other species and certainly does not extend to humans, where mutation load does not correlate with muscle fibre size (Bua *et al*. 2006; Picard *et al*. 2012). Interestingly, in inclusion body myositis, an age‐related inflammatory myopathy, respiratory‐deficient myofibres are more prone to atrophy than their unaffected counterparts (Rygiel *et al*. 2014). It is therefore difficult to conclude what the true relationship between respiratory chain dysfunction and muscle fibre atrophy is.

How prevalent are respiratory‐deficient myofibres in ageing muscle? The most comprehensive study that quantified the level of the respiratory deficiency in ageing human muscle reported up to 5% deficient fibres in 60‐ to 90‐year‐old participants (Brierley *et al*. 1996). Preliminary data obtained in our laboratory for a large cohort of 70‐year‐old participants showed 6–14% of respiratory‐deficient fibres in the most affected cases (unpublished data). The proportion of the respiratory‐deficient fibres may not appear high and their significance is questionable. It is worth stressing, however, that the deficiency is segmental and can affect any fragment of the muscle fibre along its length. The proportion of respiratory‐deficient cells is typically derived from assessment of a single or a small number of muscle sections, which may significantly underestimate the true deficiency in the entire muscle.

The next fundamental question is whether mitochondrial dysfunction has a functional consequence for muscle wasting in sarcopenia. Patients with mitochondrial disease tend to develop myopathy, involving selected muscle groups, which is mostly associated with weakness (McFarland & Turnbull, 2009). Data from animals are somewhat puzzling. A mouse model with a skeletal muscle conditional knock‐out of the *COX10* gene (encoding a subunit of mitochondrial respiratory chain complex IV) showed virtually no reduction in maximal contractile force for the first 2.5 months of age despite complex IV activity being reduced by over 95% (Diaz *et al*. 2005). Additionally, there was only a 10% increase in fatigability and no signs of oxidative damage or apoptosis in young mice, suggesting that the relationship between mitochondrial respiratory chain function and muscle phenotype is not direct (Diaz *et al*. 2005). However, the *COX10*‐induced myopathy worsened with time, consistent with progressive muscle degeneration in humans.

### Mitochondrial dynamics and degradation

In the ageing muscle, abnormal mitochondrial dynamics of fusion/fission could contribute to muscle dysfunction via two major mechanisms. The first relates to the fact that mitochondrial dynamics is essential to mitophagy and quality control processes. Fission is necessary for subsequent mitophagy to remove defective proteins or mtDNA molecules from the mitochondrial network. In this case, inhibition of fission and thus mitophagy may promote accumulation of dysfunctional mitochondria within skeletal muscle fibres (Masiero *et al*. 2009; Grumati *et al*. 2010). The down‐regulation of specific Parkin machinery responsible for culling and degrading dysfunctional mitochondria may also be down‐regulated in aged skeletal muscle (Gouspillou *et al*. 2014). Secondly, dysfunctional mitochondria may promote atrophy via multiple different pathways discussed above, including pro‐apoptotic signalling (Gouspillou *et al*. 2014), energy deficiency and impacting nuclear gene expression.

With ageing, mitochondrial dynamics is altered and mitochondria undergo structural remodelling. Electron microscopy performed on muscle samples from old mice revealed changes in both subsarcolemmal and intermyofibrillar mitochondria: in aged muscle exhibiting a 30% reduction in myofibre size, subsarcolemmal mitochondria were larger and more elongated whereas intermyofibrillar mitochondria were longer and more branched than in young muscle (Leduc‐Gaudet *et al*. 2015). Together with the up‐regulated protein ratio of Mfn2/Drp1 and larger mitochondrial volumes, this strongly suggested increased fusion in both mitochondrial subpopulations (Leduc‐Gaudet *et al*. 2015) (Fig. 3). In support of these findings, a different mouse study found up‐regulated Mfn1 and Mfn2 and down‐regulated Fis1 (Joseph *et al*. 2013), and mitochondrial elongation was observed in fibroblasts of aged individuals (Allen *et al*. 2015). Apparently in contrast to these findings, mRNA levels for Mfn2 were found to be down‐regulated in elderly human skeletal muscle (Crane *et al*. 2010). Keeping in mind that mRNA levels do not directly translate into corresponding protein levels, and that mitochondrial dynamics proteins are heavily post‐translationally regulated (Shutt *et al*. 2012), this discrepancy could be attributable to different factors and be without relevance to mitochondrial dynamics. Overall, atrophying skeletal muscles may exhibit excess mitochondrial fusion, which consists in a normal response to mild stress (Shutt & McBride, 2013), and may thus represent a compensatory response to an intrinsic functional defect within aged organelles.

Because mitochondria have to undergo fragmentation in order to allow autophagosome formation and engulfment, mitochondrial elongation via increased fusion in aged muscle could prevent degradation via autophagy (Rambold *et al*. 2011). In line with this, a general decrease in proteolysis pathways has been reported in various ageing tissues from senescent mice, rats and humans (Cuervo & Dice, 2000; Ferrington *et al*. 2005; Wohlgemuth *et al*. 2010). Data from the skeletal muscle, however, is limited and not conclusive, particularly with regard to the proteasome and mitophagy pathways. Lon protease‐associated degradation is the only system that has been clearly shown to be affected in ageing skeletal muscle (Bota & Davies, 2002). Lon protein levels are significantly down‐regulated in the muscle of old mice and cabonylated proteins accumulate within the muscle mitochondria (Bota *et al*. 2002).

### Mitochondrial biogenesis

Skeletal muscle mitochondrial mass tends to decrease with age (e.g. Short *et al*. 2005), although this may be specific to certain muscles and species (Picard *et al*. 2011). This is believed to be primarily due to reduced mitochondrial biogenesis, i.e. the production of new mitochondria. As mitochondrial proteins are encoded in both mitochondrial and nuclear genomes, biosynthesis of new organelles requires transcription factors and molecular regulators that act on them both. Peroxisome proliferator‐activated receptor γ coactivator (PGC) 1α is considered a master regulator of mitochondrial biogenesis and has been shown to be decreased at both mRNA and protein level in aged skeletal muscle (Ling *et al*. 2004; Rossi *et al*. 2009). PGC‐1α lies downstream from metabolic sensors AMP‐activated protein kinase (AMPK), sirtuin 1 (SIRT1), and mitogen‐associated protein kinase (p38MAPK), which synergize to activate PGC‐1α in the cytoplasm. This causes its nuclear and mitochondrial translocation, where it initiates expression of mitochondrial proteins by binding to the nDNA and mtDNA (Safdar *et al*. 2011). Because PGC‐1α co‐regulates several genes and its expression alone is sufficient to increase mitochondrial mass (Wu *et al*. 1999), it is considered to play an important role in skeletal muscle mitochondrial biogenesis.

However, this remains controversial since mitochondrial biogenesis can still happen in the absence of PGC‐1α. A mouse model of skeletal muscle‐specific knock‐out of PGC‐1α does not prevent exercise‐induced mitochondrial biogenesis (Rowe *et al*. 2012). Similarly, removal of PGC‐1α from cultured myoblasts using siRNA technology fails to inactivate genes involved in oxidative metabolism and mitochondrial biogenesis following forced contractile activity (Uguccioni & Hood, 2011). Currently, pathways other than PGC‐1α‐dependent are under investigation. PGC‐1β, a close homologue of PGC‐1α, has been shown to stimulate mitochondrial biogenesis in an equally robust manner, and deletion of both PGC‐1α and PGC‐1β from muscle down‐regulates mitochondrial function much more dramatically than deletion of either alone (Arany *et al*. 2007; Zechner *et al*. 2010). Another pathway reported to be critical for exercise‐induced biogenesis is mediated by p38MAPK (Pogozelski *et al*. 2009). For a more detailed discussion on this topic, we refer the reader to comprehensive reviews (Hawley *et al*. 2014; Drake *et al*. 2015).

PGC‐1α also up‐regulates mtDNA replication, transcription and stability. This is achieved by a twofold mechanism involving its direct association with the mitochondrial transcription factor A (TFAM) at the D‐loop region of mtDNA, and activation of nuclear respiratory factor 1 and 2 (NRF‐1 and NRF‐2, respectively), which stimulate expression of TFAM and its import into mitochondria, thus promoting mtDNA transcription and replication (Campbell *et al*. 2012) (Fig. 3).

Another interesting possibility beyond mitochondrial biogenesis stipulates that down‐regulation of PGC‐1α with ageing may also contribute to skeletal muscle atrophy by promoting destabilization of the neuromuscular junction and ‘denervation’ in aged skeletal muscle (Gouspillou *et al*. 2013). In parallel with ageing, PGC‐1α levels are reduced in sedentary persons compared with physically active individuals (Lanza *et al*. 2008). But endurance exercise can counteract the decline in both PGC‐1α expression and mitochondrial biogenesis, with data indicating that trained elderly individuals may even maintain the PGC‐1α mRNA content at a stable level exceeding that of their sedentary young counterparts (Lanza *et al*. 2008). This suggests a molecular avenue by which exercise could be an effective countermeasure to prevent sarcopenia by affecting both skeletal muscle and the NMJ.

## Exercise as a countermeasure for sarcopenia

Exercise has classically been divided into two major categories, endurance and resistance, representing two ends of a spectrum. Both exercise modalities offer benefits in terms of ageing muscle, as discussed elsewhere in detail (Barbieri *et al*. 2015). Briefly, to summarize their major effects, resistance exercise mainly leads to muscle hypertrophy, and increase in muscle mass and, in most cases, strength and power output (Cadore *et al*. 2014), whereas endurance exercise, or aerobic training, mainly improves cardiorespiratory fitness (maximal oxygen consumption – V˙o2 max ), muscle oxidative capacity and overall physical performance (Cadore *et al*. 2014). Another form of physical activity is high‐intensity interval training (HIT, or HIIT), which involves intermittent short bouts of maximum activity interspersed with periods of low intensity. This type of training provides similar benefits to endurance training but volume and time of exercise are markedly reduced (Little *et al*. 2010). More importantly in the context of exercise adaptations is that HIT induce molecular adaptations that trigger mitochondrial biogenesis. For instance, 2 weeks of HIT was sufficient to increase the amount of PGC‐1α in the nucleus, as well as overall SIRT1 and TFAM content, resulting in increased muscle mitochondrial mass and exercise performance, demonstrating that HIT elicits robust adaptive mitochondrial and physiological responses opposite to those associated with ageing (Little *et al*. 2010).

Exercise‐induced adaptations involve proportional changes in mitochondrial biogenesis and content. For example, across various exercise modalities exercise‐induced increases in V˙o2 max  occur in proportion to increase in mitochondrial content indexed by citrate synthase activity (Vigelso *et al*. 2014). This represents additional evidence that exercise‐induced physiological adaptations are linked, and could be dependent upon mitochondrial biogenesis.

Aerobic training can improve mitochondrial function irrespective of age, although the mechanisms may differ between young and older, and between male and female individuals (Vigelso *et al*. 2014). An acute endurance exercise programme up‐regulated proteins involved in activation of the electron transport chain components such as mitochondrial SIRT3, as well as mitochondrial antioxidant capacity, in older adults (>65 years) (Johnson *et al*. 2014). Nevertheless, elevated protein degradation and reduced oxidative damage were only observed in the young, suggesting age‐specific effects (Johnson *et al*. 2014). In contrast, 12 weeks of aerobic exercise intervention in 20‐ and 70‐year‐old participants resulted in increased aerobic capacity, skeletal muscle size and markers of mitochondrial biogenesis and dynamics in both groups (Konopka *et al*. 2014). Transcriptome (mRNA levels) analysis showed down‐regulation of mitochondrial genes and pathways engaged in oxidative phosphorylation with ageing, whereas redox homeostasis genes were up‐regulated in older sedentary adults. Interestingly, those differences were not present between chronically endurance‐trained older adults and their young counterparts (Johnson *et al*. 2014), suggesting that exercise is an effective countermeasure against ageing‐associated transcriptional remodelling.

Endurance exercise may also impact muscle function via epigenetic effects, consisting of modifications of DNA‐associated proteins and the DNA itself (i.e. the chromatin) that promote stable changes in gene expression (Bird, 2007). One such modification is DNA cytosine methylation, which was found to be decreased overall in skeletal muscle samples from sedentary men and women following acute exercise (Barres *et al*. 2012). Exercise induced a dose‐dependent expression of key metabolism regulator genes such as PGC‐1α, PDK4 and PPAR‐δ, with corresponding hypomethylation of their respective promoter regions, consistent with exercise‐induced epigenetic regulation of gene transcription (Barres *et al*. 2012).

Perhaps the most valuable data on the effects of life‐long endurance training on muscle function come from a study on over‐80‐year‐old athletes (Trappe *et al*. 2013). Muscle biopsy analyses revealed a markedly higher oxidative profile consisting of elevated mitochondrial markers in athletes compared with their untrained peers. Moreover, the magnitude of differences in mitochondrial function between the trained and untrained octogenarians was comparable to the differences between trained and untrained young adults (Burgomaster *et al*. 2008). Importantly, commencing endurance training after the age of 80 did not improve the age‐related decline in mitochondrial function, suggesting that only life‐long regular exercise results in the metabolic flexibility necessary to maintain a healthy muscular system (Trappe *et al*. 2013).

In light of the above, it appears difficult to discriminate between the role of ageing and inactivity on skeletal muscle deterioration. It is apparent that sedentary behaviour accelerates age‐related decline in muscle structure and function (Booth *et al*. 2011). Evidence collected so far suggests that a life‐long regular exercise can significantly delay the onset of sarcopenia (Barbieri *et al*. 2015).

## Conclusion

The human motor system deteriorates with age, leaving no one untouched. However, the rate of this deterioration varies from person to person. The underlying mechanisms are still unclear, but it has become clear that changes among both the nervous and the muscular systems are implicated in this degenerative process. It is likely that individual factors predispose to sarcopenia, for malfunction of one or the other, which makes our neuromuscular system age differently. There are also important inter‐individual differences in levels of physical activity and sedentary behaviour, diet and other factors, making experimental discrimination of various contributing factors difficult. Most likely, there are different pathways or ‘trajectories’ of ageing that are impacted by a variety of personal and environmental factors (Picard, 2011).

Mitochondria, as essential powerhouses and signalling organelles, are implicated in sarcopenia. Experimentally, mitochondrial abnormalities have been identified in both neurons and muscle fibres in elderly and sedentary subjects, and known mechanisms exist whereby abnormal mitochondrial functions can promote neuromuscular disorders. However, it is still unclear whether mitochondrial dysfunction, at a level reported for these two tissues in normal human ageing, is a primary cause of the phenotypic and functional changes seen in sarcopenia. Nevertheless, together with other dysregulated processes involved in sarcopenia, mitochondrial abnormalities are likely to contribute to loss of skeletal muscle mass and function with age (Hepple, 2014). Technical advances to probe mitochondria at a molecular level, longitudinal studies in humans, and comprehensive hypotheses involving both nerve and muscle factors should enhance our ability to understand and prevent sarcopenia.

## Additional information

### Competing interests

None declared.

### Author contributions

All authors have approved the final version of the manuscript and agree to be accountable for all aspects of the work. All persons designated as authors qualify for authorship, and all those who qualify for authorship are listed.

### Funding

The authors’ work is supported by the Newcastle University Centre for Ageing and Vitality (supported by the Biotechnology and Biological Sciences Research Council and Medical Research Council [G016354/1]), Wellcome Trust Centre for Mitochondrial Research [G906919], MRC Centre for Neuromuscular Disease [G000608‐1], The MRC Centre for Translational Research in Neuromuscular Disease Mitochondrial Disease Patient Cohort (UK) [G0800674], The Lily Foundation, and the UK NIHR Biomedical Research Centre in Age and Age Related Diseases award to the Newcastle upon Tyne Hospitals NHS Foundation Trust.
